# Pulmonary fibrosis and microscopic polyangiitis in a 75-year-old woman

**DOI:** 10.31138/mjr.30.1.44

**Published:** 2019-03-28

**Authors:** Sofia Koutsoviti, Antonia Elezoglou, Chaido Katsimpari, Ioannis Sofianos, Ioannis Raftakis, Evangelos Theotikos, Charilaos Samaras, Ioannis Myriokefalitakis

**Affiliations:** 1 Rheumatology Department of Asklepieion Voulas General Hospital, Athens, Greece,; 2 1^st^ Internal Medicine Department of Asklepieion Voulas General Hospital, Athens, Greece

**Keywords:** pulmonary fibrosis, microscopic polyangiitis, anti-MPO p-ANCA

## Abstract

We present a case of a 75-year-old woman who admitted in the internal medicine department for a recent onset of persisting moderate daily fever and fatigue that started 30 days prior to her hospitalization. Her past medical history is remarkable for mild pulmonary fibrosis, megaloblastic anaemia, and hypergammaglobulinaemia of no obvious causes. On presentation, she was febrile (38°C) and had high ESR and CRP levels, but most of her laboratory tests were within normal levels and had no signs of arthritis or rash. She was hospitalized for suspected lower urinary tract infection and started on antibiotics. During hospitalization, her renal function deteriorated together with microscopic haematuria, proteinuria and granular urine casts in urine analysis and her inflammation markers raised further. A renal biopsy revealed glomerulonephritis with pauci-immune crescents, and serology tests were positive for anti-MPO p-ANCA, both suggesting a diagnosis of microscopic polyangiitis (MPA). While high-dose methylprednisolone pulses and cyclophosphamide were introduced intravenously, there was no remission, but respiratory failure occurred that led to patient’s intubation and transfer to the ICU. She died a few days later due to septic shock. Asymptomatic pulmonary fibrosis can precede microscopic polyangiitis for several years and is associated with a poor prognosis.

## INTRODUCTION

Microscopic polyangiitis (MPA) is an anti-neutrophil cytoplasmic antibodies (ANCA) associated vasculitis, affecting the small vessels, capillaries or arterioles, that is characterized by few or no immune deposits and the absence of granulomas on histopathological specimens.^1^ It is a rare disease entity and can affect all racial groups, with predominance in Caucasians. Male-to-female ratio is 1,1:1,8 and the average age at onset is 50 years.^1^ Clinical manifestations vary between mild general symptoms such as fever, myalgias and arthralgias, and serious renal, nervous and lung involvement. The characteristic pulmonary clinical feature is the diffuse alveolar haemorrhage due to pulmonary capillaritis. Interstitial lung fibrosis is a less-recognized manifestation which may or may not follow alveolar haemorrhage. It has also been described as an isolated process in some patients with positive anti-myeloperoxidase perinuclear anti-neutrophil cytoplasmic antibodies (anti-MPO pANCA). We present a patient with a rapidly progressive glomerulonephritis whose diagnosis of lung fibrosis predated the vasculitis for five years.

## CASE DESCRIPTION

A 75-year-old woman with a history of mild pulmonary fibrosis, megaloblastic anaemia and hypergammaglobulinaemia was hospitalized for a recent onset of moderate fever (up to 38°C twice a day), dry cough and fatigue in the 1^st^ internal medicine department at our hospital. Her symptoms started a month prior to her hospitalization. Her asymptomatic pulmonary fibrosis was revealed in a routine chest X-ray examination 5 years ago. On that occasion, a chest computerized tomography (CT) scan was ordered by a pulmonologist and the interstitial pattern was compatible with pulmonary fibrosis. During follow-up her condition remained stable, with no clinical signs of dyspnoea, or shortness of breath or increased inflammation markers. She also suffered from megaloblastic anaemia treated with intra-muscular (i.m.) hydroxocobalamin. In addition, she was diagnosed nine months ago with a monoclonal IgG-λ-type hypergammaglobulinaemia and had a positive anti-nuclear antibody (ANA) titre of 1/640 (diffuse pattern). No further investigation was made at that time. During her hospitalization, erythrocyte sedimentation rate (ESR) and C-Reactive Protein (CRP) levels reached a peak of 125 mm (normal range: 0-20 mm) and 98 mg/l (normal range: 0–5 mg/l) respectively, and a positive urine culture for *Proteus mirabilis* was suggestive of a lower urinary tract infection. She started on ceftriaxone intravenously (i.v.), but still remained febrile after the first week of her treatment. An extensive work-out was performed to rule out tuberculosis (TBC), endocarditis, and other atypical infections, performed with multiple blood and sputum cultures, virology tests for Hepatitis B virus (HBV), Hepatitis C virus (HCV), Ebstein-Barr virus (EBV), heart ultra-sonography studies, upper and lower gastrointestinal endoscopy and abdomen CT scans that were all unremarkable, without any suggestive cause. Chest CT scans only revealed her previously known stable mild pulmonary fibrosis, and bone marrow examination revealed plasma cells less than 5%.

On the 10^th^ day of hospitalization, while the patient was still febrile, a decline of her renal function was present with high creatinine (Cr) and urea serum levels at 2.3mg/dl (normal range 0.5–1.5 mg/dl), and 83mg/dl (normal range 10–50 mg/dl) respectively and mild proteinuria (protein: 1+), moderate haematuria (Hb: 3+) with the presence of bacteria in the urine analysis. Moreover, mild leucocytosis 10.400k/μl (normal range 4.60–10.20 k/μl) and prominent anaemia (Hemoglobin: 9.1g/dl [normal range: 12.2–18.1 g/dl], Haematocrit: 28.1% [normal range: 37.7–53.7%]) were now present. Her treating physician requested a rheumatological consultation on signs of inflammatory connective tissue disease. On evaluation she had a fever of 38°C, end-inspiratory bibasal crackles, normal arterial blood pressure and heart rate. She had no rash, no palpable purpura, livedo reticularis or Raynaud phenomenon, neither any sign of arthritis, nor any history of photosensitivity, hair loss, leukopenia or cryoglobulinemia.

A full immunology work-out was performed, the patient was admitted to our department and methylprednisolone 16 mg/day was administered on top of antibiotics with close monitoring of *Proteus mirabilis* lower urinary tract infection and renal function.

**Figures 1a and 1b.**
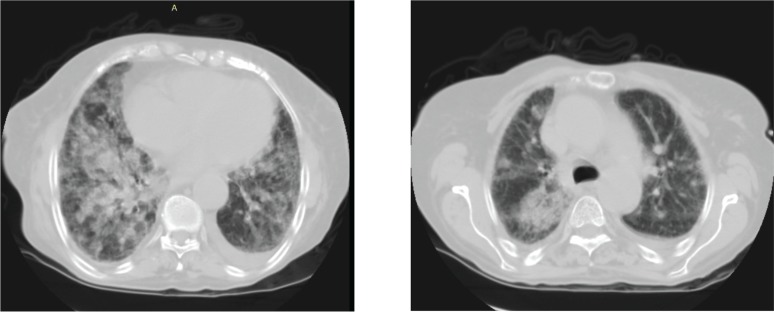
Diffuse bilateral pulmonary consolidation, thickening of bronchovascular bundles and ground glass opacity.

Even though there was a slight improvement with a drop in temperature (37.4°C) and creatinine serum levels (1.6 mg/dl) right after the initiation of methylprednisolone, a relapse occurred (Cr: 2.5mg/dl, urea: 126 mg/dl), a week later with prominent proteinuria (1.72 g/24h) and the presence of red blood cells (50), white blood cells (15–20) and granular urine casts at urine analysis, suggesting the immediate need for a renal biopsy. The renal histopathology revealed pauci-immune glomerulonephritis with crescentic formation, and perinuclear anti-MPO pANCA (in high levels) were present in the patient’s serum, both suggesting a certain diagnosis of microscopic polyangiitis. Antinuclear antibodies (ANA) were also present in the moderate titre of 1/320, whereas anti-double stranded DNA (anti-dsDNA), anti-extractable nuclear antigen (anti-ENA) and anti-cardiolipin antibodies were all absent. Treatment initiation with three daily pulses of 1gr methylprednisolone was introduced together with trimethoprim/sulfamethoxazole prophylaxis, following by a pulse dose of cyclophosphamide adjusted to patient’s renal impairment (GFR 14.2 ccs/min). On the day of the cyclophosphamide pulse, the patient deteriorated with dyspnoea, haemoptysis and quickly developing respiratory failure. An immediate CT scan revealed newly scattered lung infiltrates and ground glass areas, attributed to pulmonary haemorrhage (Figure 1a and Figure 1b),, whereas the blood gas analysis showed pCO2 30mmHg, pO2 62mmHg, pH 7,25, sO2 87% (FiO2 100%), suggesting to patient’s intubation and transfer to the ICU. In the following days, her renal function got worse (Cr 4mg/dl, urea 320mg/dl) and renal haemodialysis was performed. She finally died 13 days later in the course of septic shock (Hypotension, WBC 24.900 K/μl, Hb 8,2 g/dl, PLT 37.4 K/μl Cr 2,7 mg/dl, TBil 2,4 mg/dl).

## DISCUSSION

Microscopic polyangiitis is a rare ANCA-associated vasculitis, and diffuse alveolar haemorrhage is its hallmark pulmonary manifestation. Hence, its association with pulmonary fibrosis has been increasingly reported in recent literature, even as a possible first manifestation of MPA, and is associated with increased mortality.^1,7,8^ Respiratory symptoms related to fibrosis may precede other manifestations by a median period of 13 (5–120) months.^3–6^ Some data also imply that there is a causal relationship between idiopathic interstitial pneumonia (IIP) and ANCA. The first assumption is that MPO may play a role in pulmonary fibrosis and the second that pulmonary fibrosis may induce the production of the antibody.^9–11^ Extra pulmonary manifestations in IPF requires ANCA testing in order to rule out the possibility of a co-existing vasculitis. IPF with positive anti-MPO antibodies is associated with the risk of MPA development and certainly worst prognosis.^12^

## ABBREVIATIONS

ANA: anti-nuclear antibodies
anti-dsDNA: anti-double stranded DNA
anti-ENA: anti-extractable nuclear antigen
anti-MPO p-ANCA: anti-myeloperoxidase perinuclear anti-neutrophil cytoplasmic antibodies
CRP: C-Reactive Protein
CT: Computerized Tomography
EBV: Ebstein-Barr virus
ESR: Erythrocyte Sedimentation Rate
i.m.: intra-muscular
i.v.: intravenously
ICU: Intensive Care Unit
IPF: Interstitial Pulmonary Fibrosis
HBV: Hepatitis B virus
HCV: Hepatitis C virus
IIP: Idiopathic Interstitial Pneumonia
MPA: Microscopic Polyangiitis
TBC: tuberculosis
